# RNF8 depletion attenuates hepatocellular carcinoma progression by inhibiting epithelial-mesenchymal transition and enhancing drug sensitivity: The role of RNF8 in EMT and drug sensitivity in HCC

**DOI:** 10.3724/abbs.2023076

**Published:** 2023-05-06

**Authors:** Jingyu Kuang, Ting Duan, Changsong Gao, Chuanyang Liu, Si Chen, Lv-yun Zhu, Lu Min, Chenyu Lu, Wenlun Wang, Lingyun Zhu

**Affiliations:** 1 Department of Biology and Chemistry College of Sciences National University of Defense Technology Changsha 410073 China; 2 School of Pharmacy Hangzhou Normal University Hangzhou 311121 China; 3 Department of Pathology Hunan Provincial People’s Hospital Changsha 410073 China

**Keywords:** hepatocellular carcinoma, epithelial-mesenchymal transition, RNF8, drug resistance

## Abstract

Despite substantial advances that have been made in understanding the etiology of hepatocellular carcinoma (HCC), the early-stage diagnosis and treatment of advanced-stage HCC remain a major challenge. RNF8, an E3 ligase important for the DNA damage response, has been proven to facilitate the progression of breast and lung cancer, but its role in HCC remains unclear. In this study, we find that the expression of RNF8 is up-regulated in HCC tissues and positively correlated with poor prognosis of HCC. Furthermore, silencing RNF8 by siRNAs attenuates the migration of HCC cells and inhibits epithelial-mesenchymal transition (EMT) by regulating the expressions of proteins including N-cadherin, β-catenin, snail, and ZO-1. Moreover, Kaplan‒Meier survival analysis shows that high RNF8 expression predicts poor survival benefits from sorafenib. Finally, cell viability assay demonstrates that RNF8 depletion enhances the sensitivity of HCC cells to sorafenib and lenvatinib treatment. We hypothesize that the inhibitory role of RNF8 in EMT and its enhancing effects on anti-cancer drugs orchestrate the protective effects of RNF8 deficiency in HCC, which indicates its potential in clinical application.

## Introduction

Primary liver cancer is one of the most common malignancies worldwide, ranking as the sixth most prevalent and the third most lethal cancer, with approximately 906,000 new cases and 830,000 deaths in 2020 [1]. Hepatocellular carcinoma (HCC) is the dominant type of hepatic cancer that originates from hepatocytes, and it is estimated that HCC constitutes 75%‒85% of primary liver tumors [2]. Despite significant advances in elucidating the etiology and exploiting novel therapeutic strategies, the 5-year survival rate of HCC remains very low, especially for advanced HCC patients. It has been estimated that the 1-year survival rate for HCC is 60.5%, and the 3-year survival rate is 27.6%, while only 8.37% of HCC patients survive beyond five years [3].

Management of advanced HCC with inoperable tumors remains challenging, and systemic chemotherapy is the preferred choice for those patients. Sorafenib, an oral multikinase inhibitor against receptor tyrosine kinases such as VEGFR, is the first targeted drug approved by the U.S. Food and Drug Administration (FDA) for first-line systemic therapy [4]. Despite its side effects, sorafenib has been the unique target drug for advanced HCC for over a decade. Lenvatinib, another oral multikinase inhibitor targeting VEGFR1-3, FGFR1-4, PDGF, RET, and KIT, was approved by the FDA for the first-line systemic treatment of patients with unresectable HCC in 2018. Compared with sorafenib, lenvatinib showed similar efficacy but less palmar-plantar erythrodysesthesia [5]. Later, agents including cabozantinib, regorafenib, ramucirumab, and nivolumab were approved by the FDA for second-line systemic treatment to treat patients who tolerated sorafenib or lenvatinib [6]. Although numerous improvements have been made in HCC treatment, sorafenib and lenvatinib are still the up-front choices for advanced HCC and have been proven to increase the survival of those patients [7]. However, for advanced-stage HCC patients with metastasis or drug resistance, these therapies have shown limited effectiveness, which suggests the urgent need for methods to combat metastasis and drug resistance in liver cancer.

The really interesting new gene (RING) finger protein 8 (RNF8) is a member of the RING finger family and functions as an E3 ligase that plays an essential role in double-strand break (DSB) repair [8]. RNF8 contains two conserved domains: the N-terminal forkhead-associated (FHA) domain that can specifically dock the ligase to target proteins, and the RING-finger motif which is responsible for ubiquitin ligase activity [9]. In the context of DNA damage, H2AX (histone H2A variant) is phosphorylated by activated ATM (ataxia-telangiectasia mutated), followed by MDC1 (mediator of DNA damage checkpoint 1) interaction and subsequent phosphorylation of MDC1 [10]. RNF8 recognizes phosphorylated MDC1 at DSB sites and catalyzes K63 ubiquitination of histone H2A and H2AX by cooperating with UBC13, which promotes the recruitment of RNF168 [ 11, 12] . Ubiquitination of histones at DSBs promotes the recruitment of other repair proteins, including BRCA1 and 53BP1, and these key proteins work together to propagate the DSB damage repair process [13]. In addition to the classic role of RNF8 in DNA damage repair, RNF8 also participates in telomere protection, cell cycle regulation, and transcriptional regulation [14]. Concerning its critical role in DNA damage, RNF8 has been regarded as a tumor suppressor for a long time [15]. However, recent studies have reported that RNF8 might promote tumor progression in colorectal cancer and nasopharyngeal cancer [ 16, 17] .

Previously, we discovered that RNF8 promoted epithelial-mesenchymal transition in breast cancer and lung cancer [ 18, 19] , which indicated the potential role of RNF8 in cancer metastasis. However, the function of RNF8 in hepatocellular tumorigenesis remains unknown despite reports that RNF8-induced K63 ubiquitination of Twist1 is involved in the trabid-mediated inhibition of HCC growth and metastasis [20]. In this study, based on clues from public databases showing up-regulated expression of RNF8 in HCC tissues and its relationship with poor prognosis in liver cancer, we performed experiments in HCC cell lines and patient tissues. We confirmed the higher expression of RNF8 in HCC cells and tissues and further revealed that RNF8 deficiency exerted an inhibitory effect on HCC by regulating cell migration, EMT and drug sensitivity to sorafenib and lenvatinib, which holds promise for its clinical application in HCC patients.

## Materials and Methods

### Tissues

Tumor and adjacent non-tumor tissues were collected from 17 HCC patients who underwent surgical resection at Hunan Provincial People′s Hospital (Changsha, China). Written informed consent was obtained from each participating patient and the study was approved by both the Ethical Board of Hunan Provincial People′s Hospital and the Institutional Ethical Board of the College of Science, National University of Defense Technology (Changsha, China). The clinical information of HCC samples were listed in Supplementary Table S1.

### Cell culture

The human hepatic normal cell line HL7702 and liver cancer cell lines QGY-7703 and SMMC7721 were purchased from Procell Life Science & Technology Co., Ltd. (Wuhan, China ), and cultured in high glucose DMEM (HyClone, Logan, USA) containing 10% FBS. The liver cancer cell line HepG2 was purchased from the National Collection of Authenticated Cell Cultures (Beijing, China) and cultured in RPMI 1640 (HyClone) supplemented with 10% FBS (HyClone). Human liver cancer cell lines, including SNU387, Huh7, and HCCLM3 cells, were obtained from ATCC (Manassas, USA) and cultured in high glucose DMEM (HyClone) containing 10% FBS. All cell lines were maintained at 37°C with 5% CO _2_ in a humid atmosphere. Cell lines were tested for mycoplasma contamination using polymerase chain reaction.

### siRNA sequences and transfection

siRNAs against RNF8 were synthesized by GenePharma (Shanghai, China), and the sequences are listed below: siRNF8-1 (5′-GGACAAUUAUGGACAACAA-3′); siRNF8-2 (5′-UGCGGAGUAUGAAUAUGAA-3′); siControl (5′-UUCUCCGAACGUGUCACGUTT-3′). Transient transfections were performed using Lipofectamine® RNAiMAX Reagent (Thermo Fisher, Waltham, USA) according to the manufacturer’s instructions.

### Western blot analysis

Protein expression was detected by western blot analysis as described previously [19]. Briefly, RIPA buffer with 1× protease inhibitor cocktail (Roche, Basel, Switzerland) was used to lyse cells. Equal amounts of protein were separated by SDS‒PAGE, and transferred to polyvinylidene fluoride (PVDF) membranes (Millipore, Billerica, USA). Membranes were blocked with 5% nonfat powdered milk (Sangon Biotech, Shanghai, China) in Tris-buffered saline containing 0.1% Tween-20 (TBST), incubated with the primary antibodies including anti-RNF8 (Santa Cruz Biotech, Santa Cruz, USA), anti-phospho-β-catenin (Cell Signaling Technology, Beverly, USA) and anti-β-actin (Santa Cruz Biotech), followed by incubation with the corresponding secondary antibodies (HRP-conjugated anti-mouse IgG or anti-rabbit IgG (Fc); ZSGB-Bio, Guangzhou, China). An epithelial-mesenchymal transition antibody sample kit (Cell Signaling Technology) was used to detect EMT-related proteins. The immunoreactive bands were visualized using an ECL detection system (Millipore). All experiments were repeated with three independent replicates.

### Cell viability

Cell Counting Kit-8 (CCK-8) assay was performed to determine cell viability. Cells were seeded in 96-well plates (2500 cells/well for HCCLM3, Huh7 and SNU387, 3000 cells/well for HepG2), treated with vehicle, sorafenib (BAY 43-9006; Selleck, Huston, USA) or lenvatenib (E7080; Selleck) for 24, 48 and 72 h. Then, 10 μL of CCK8 reagent (Dojindo, Rockville, USA) was added to each well, suspended in 100 μL complete medium and incubated for 2 h. The absorbance was measured at 450 nm.

### Migration assays

Cell migration was measured using transwell chambers with 8-μm membrane filters (Corning Co, Corning, USA). First, single cell suspensions (1×10 ^5^ for HepG2 cells, 1×10 ^6^ for HCCLM3 cells) in serum-free DMEM were added into the upper chamber, and DMEM containing 10% fetal bovine serum was added to the lower chamber. After incubation for 24 h, the cells were gently washed with PBS and fixed with 4% paraformaldehyde. Then, the non-migrated cells were scraped off with a cotton swab, and cells that migrated to the lower side from the upper chamber were stained with crystal violet. Finally, cells per microscopic field were imaged and counted in 8 random fields. Triplicate wells were performed in each assay, and the experiment was repeated independently for at least three times.

### Coimmunoprecipitation assays

The experiment was performed under the standard procedure as previously described [19]. Briefly, HEK293T cells were transfected with the corresponding plasmid TG006 (From Dr. Genze Shao, School of Basic Medical Sciences, Peking University Health Science Center, Beijing, China) or TG006-FH-RNF8, adapted to suspension conditions and lysed in NETEN buffer with 1 mM PMSF and protease inhibitor cocktail after 48 h of culture. The supernatants were then incubated with anti-HA-Tag-Agarose beads (Abmart, Shanghai, China) for 4 h at 4°C, and the bound proteins were analysed by western blot analysis.

### Statistical analysis

Data are presented as the mean±SEM. An unpaired two-sided Student’s *t* test was used to compare differences between two independent groups, except where indicated otherwise. Kaplan‒Meier survival analysis for the relationship between survival time and the RNF8 signature or between RNF8 expression and sorafenib benefit in HCC was performed using an online tool ( http://kmplot.com/analysis/). LIHC pancancer normalized mRNA expression data were downloaded from the UCSC Xena datahub, and the ″limma″ package in R was used to perform differential expression analysis. Genes with log2 (fold change)>0.5 and adjusted *P* value<0.05 were regarded as differentially expressed genes. The expression of RNF8 was also shown using a box plot based on ″ggplot2″, and the difference between the two groups was tested by the Wilcoxon rank sum test.

## Results

### RNF8 is upregulated in HCC and positively related to poor prognosis

**Figure 1:**
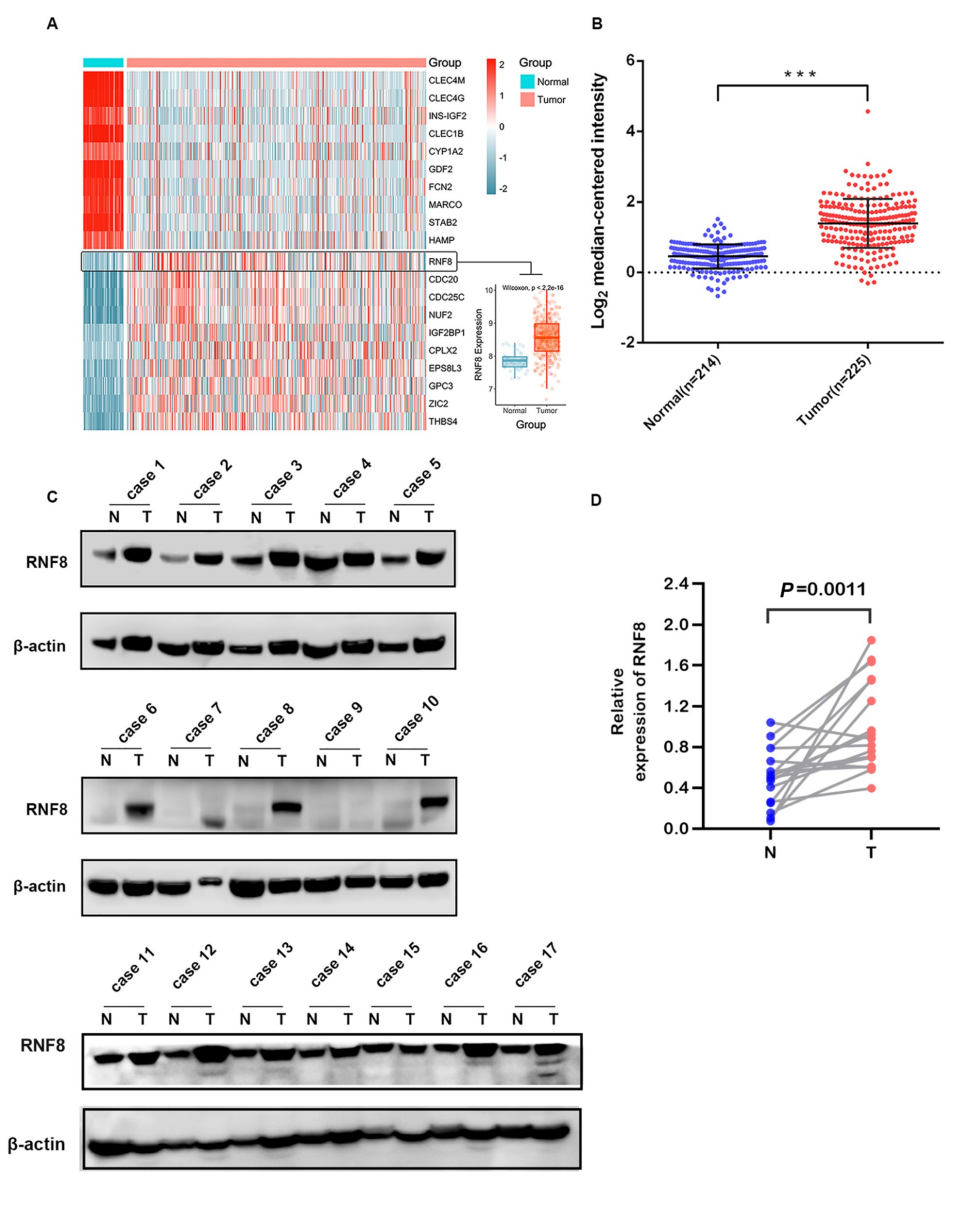
Figure 1 RNF8 was overexpressed in HCC patients (A) Heatmap showing the expressions of differentially expressed genes in liver tumor tissues ( n=373) and normal tissues ( n=50) using data from UCSC Xena. (B) Scatterplot analysis of RNF8 expression in tumor and normal tissues with data from TCGA. Western blot analysis (C) and quantification (D) of RNF8 protein in liver tumor tissue and paired adjacent non-tumor tissue. N: adjacent non-normal; T: tumor. *** P<0.001.

**Figure 2:**
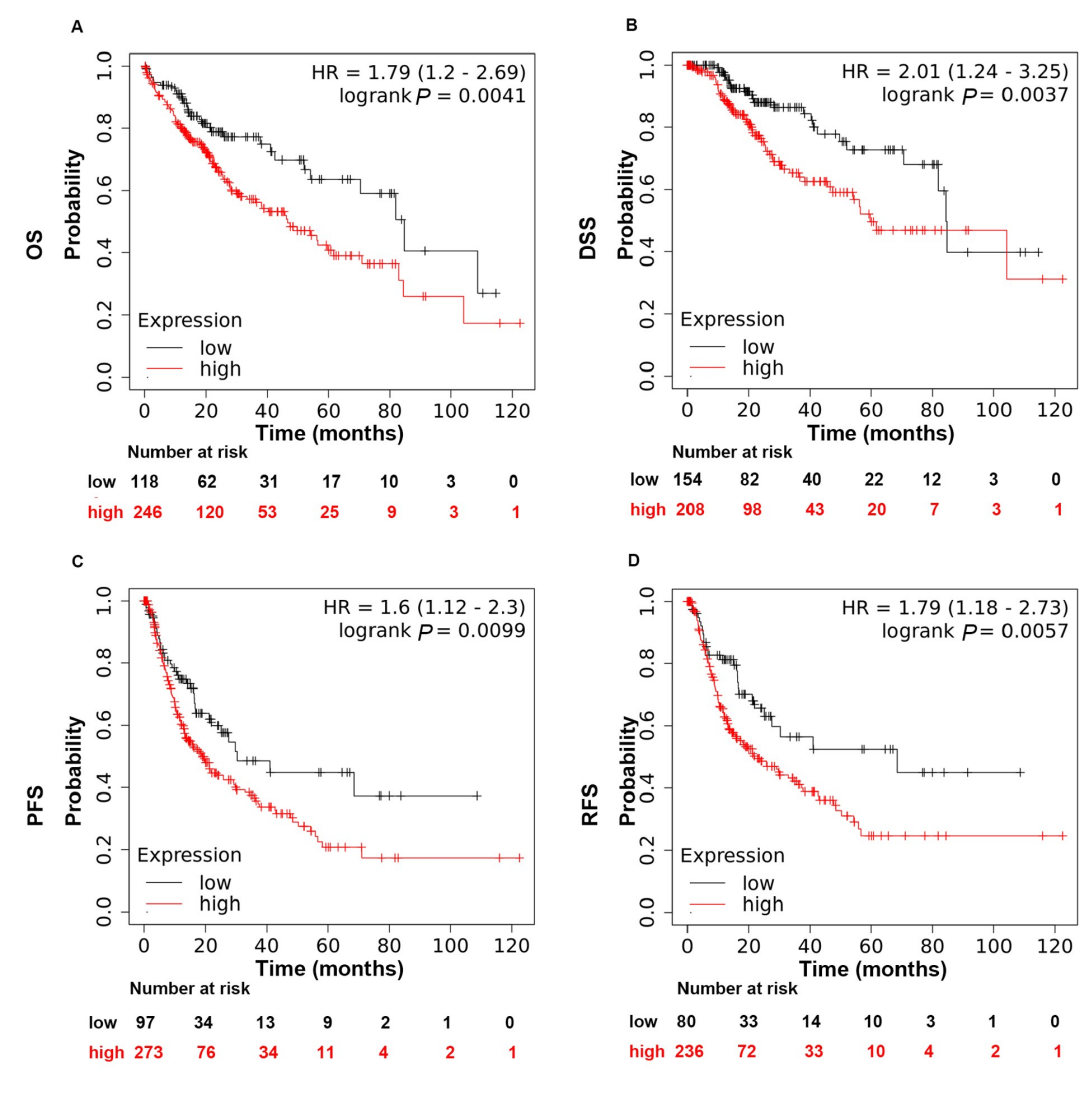
Figure 2 Expression of RNF8 in liver cancer was negatively correlated with survival time Kaplan‒Meier survival analysis for the relationship between survival time and RNF8 signature in liver cancer was performed by the online tool (http://kmplot.com/analysis/). (A) OS, overall survival. (B) DSS, disease-specific survival. (C) PFS, progression-free survival. (D) RFS, relapse-free survival. P<0.05 was considered statistically significant.

To understand the role of RNF8 in HCC, we performed differential expression analysis with data from the UCSC Xena datahub, and the heatmap showed that RNF8 was one of the best differentially expressed genes that was upregulated in liver tumor tissues compared with normal tissue ( Figure 1A). This finding was further validated by results from the Oncomine database showing that liver tumors exhibited significantly higher expression of RNF8 than normal tissues ( Figure 1B). Consistent with findings from the public database, western blot analysis of clinical HCC tissues demonstrated higher expression of RNF8 in hepatic tumors than in paired adjacent-nontumor tissues ( Figure 1C,D). It was also found that RNF8 protein was more abundant in liver cancer cell lines than in the hepatic normal cell line ( Supplementary Figure S1). We then determined the correlation of RNF8 expression with the prognosis of HCC patients using the online tool Kaplan‒Meier Plotter ( http://kmplot.com/analysis/). Kaplan‒Meier survival analysis showed that higher RNF8 expression was associated with worse overall survival (OS, *P*=0.0041), worse disease-specific survival (DSS, *P*=0.0037), worse progression-free survival (PFS, *P*=0.0099) and worse relapse-free survival (DFS, *P*=0.0057) in liver cancer patients ( Figure 2). Taken together, these findings suggested that RNF8 might play a critical role in hepatic tumor progression.

### Silencing RNF8 inhibits migration and EMT in HCC cells

**Figure 3:**
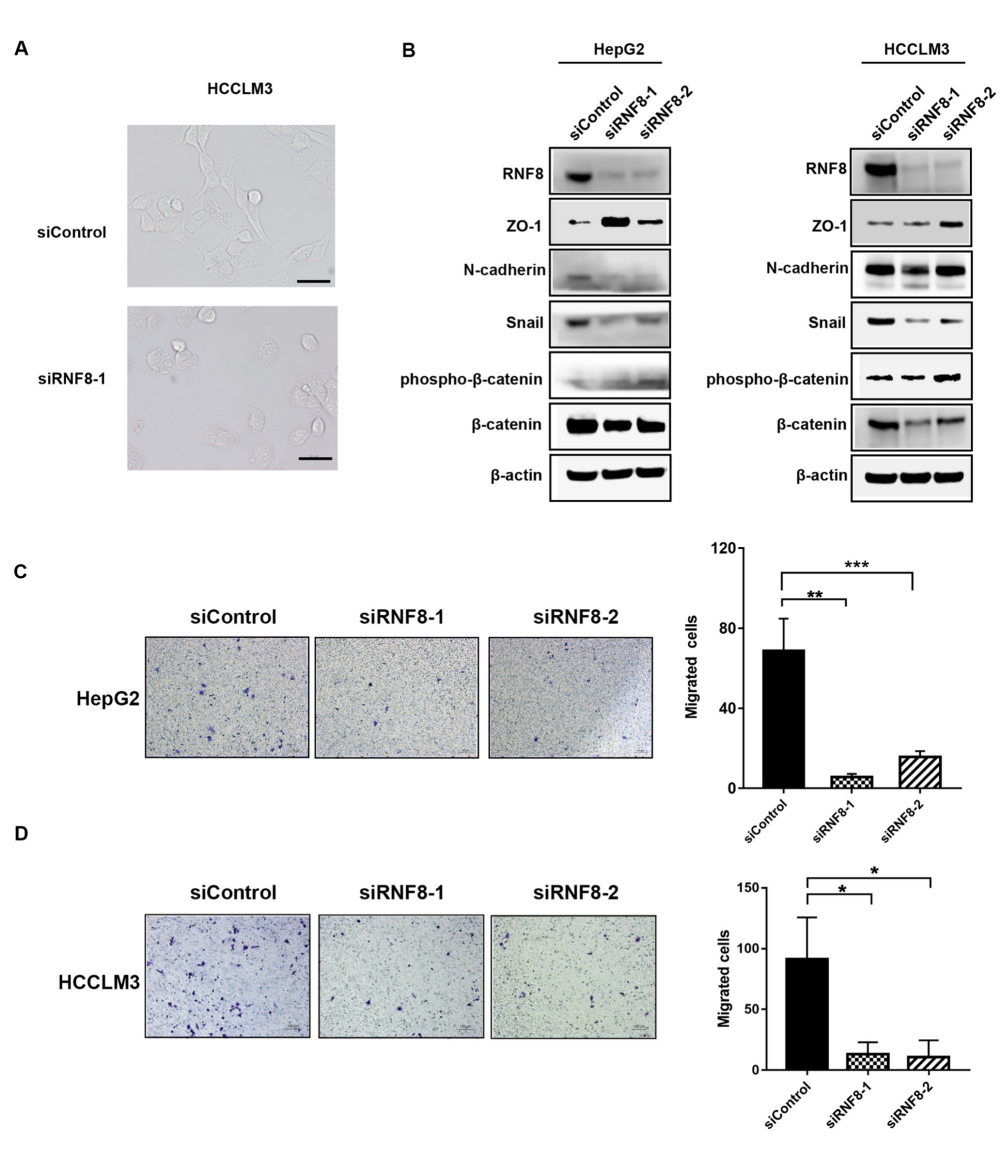
Figure 3 Silencing of RNF8 suppressed the migration and EMT of HCC cells (A) Representative morphologic images of HCCLM3 cells 72 hours after transfection with siRNA targeting RNF8. (B) EMT markers in HepG2 and HCCLM3 cells were monitored by western blot analysis 72 hours after transfection with siControl or siRNF8. Cell migration of HepG2 (C) and HCCLM3 (D) cells was detected by transwell assays 48 hours after transfection with siControl or siRNF8 ( n=3). * P<0.05, ** P<0.01, *** P<0.001.

Epithelial-mesenchymal transition (EMT) is a critical driver of cancer metastasis, which is closely associated with tumor progression [21]. Signaling such as Wnt/β-catenin leads to the activation of EMT-promoting transcription factors (TFs), including snail and slug, and those TFs coordinate the EMT process by regulating many target genes in relation to EMT [22]. First, we evaluated the effects of RNF8 on the EMT process in HCC cells. As expected, silencing RNF8 in HCCLM3 cells resulted in remarkable changes in morphology with a shape change from polygons to rounds, indicating the transition of cells from mesenchymal to epithelial ( Figure 3A). Consistent with morphological transformation, RNF8 deficiency decreased the expressions of N-cadherin, β-catenin, and snail and increased the expression of ZO-1 in HepG2 and HCCLM3 cell lines, suggesting the potential function of RNF8 in EMT ( Figure 3B). In addition, transwell assays further confirmed that knockdown of *RNF8* inhibited cell migration in HepG2 ( Figure 3C) and HCCLM3 cells ( Figure 3D). Considering the key role of RNF8 in ubiquitination as an E3 ligase, we further explored the interaction of RNF8 with EMT regulators. The results from coimmunoprecipitation revealed that ectopic RNF8 interacted with endogenous β-catenin ( Supplementary Figure S2). Thus, we proposed that RNF8 regulates EMT by affecting the expression of EMT regulators at the transcriptional or posttranslational level. Taken together, these results indicated that silencing of *RNF8* inhibited EMT and the migration capacity of liver cancer cells.

### High RNF8 expression level predicts poor sorafenib survival benefit in HCC patients

**Figure 4:**
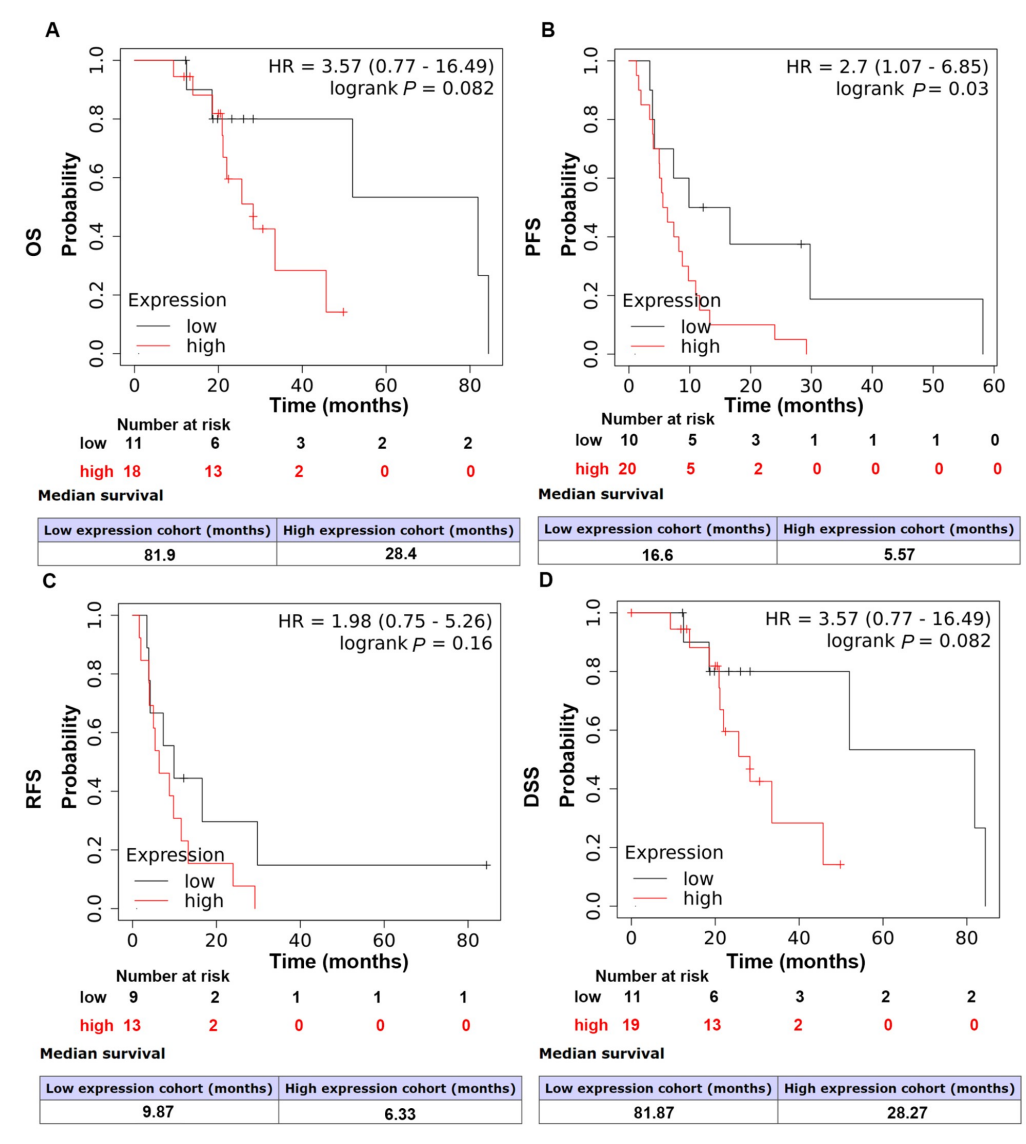
Figure 4 High RNF8 expression was associated with poor therapeutic benefits of sorafenib in HCC patients HCC patients treated with sorafenib were stratified into subgroups according to RNF8 levels and subjected to Kaplan‒Meier survival analysis. (A) OS, overall survival. (B) PFS, progression-free survival. (C) RFS, relapse-free survival. (D) DSS, disease-free survival. P<0.05 was considered statistically significant.

Drug resistance remains the principal impediment in cancer treatment and is responsible for up to 90% of cancer-related deaths [23]. Recently, EMT has received increasing attention for its role in cancer drug resistance. Based on the existing information, we wondered whether RNF8 could alter therapeutic effects in liver cancer. Thus, survival analysis was performed to uncover the correlation between RNF8 expression and sorafenib benefit. The results demonstrated that high RNF8 expression suggested poor PFS ( *P*=0.03) to sorafenib, while the analysis of OS ( *P*=0.082), RFS ( *P*=0.16) and DSS ( *P*=0.082) showed no statistical significance ( Figure 4), which was probably due to limited number of cases. The impacts on PFS further supported the significance of RNF8 in tumor progression.

### RNF8 depletion enhances the therapeutic effects of sorafenib in HCC cell lines

**Figure 5:**
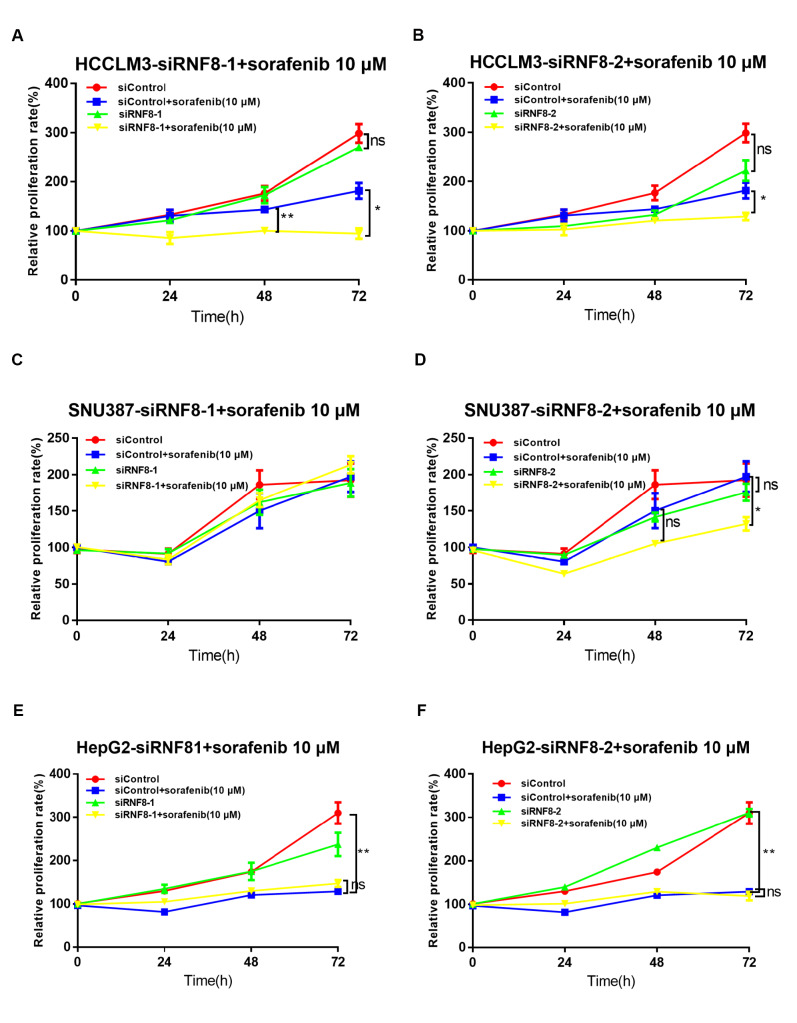
Figure 5 RNF8 depletion enhanced the drug sensitivity of HCC cells to sorafenib Cell proliferation was evaluated via CCK8 assay to detect the effects of RNF8 silencing on sorafenib efficiency in HCCLM3 (A,B), SNU387 (C,D), and HepG2 (E,F) cells. HCC cells were transfected with the indicated siRNAs and subsequently treated with sorafenib for 24, 48, and 72 h. * P<0.05, ** P<0.01, ns, no significance.

As the KM plot predicted the relationship between RNF8 and sorafenib benefit, we further confirmed its effects by *in vitro* experiments. First, we characterized sorafenib sensitivity in HCC cell lines and found that sorafenib showed the best efficiency in Huh7 cells, good efficiency in HepG2 cells, poor efficiency in HCCLM3 cells, and the worst efficiency in SNU387 cells ( Supplementary Figure S3). We then examined the effects of *RNF8* silencing on sorafenib efficiency in liver cancer cells. Huh7 cells displayed overwhelming cell death when subjected to a combination treatment of sorafenib and RNF8 depletion (data not shown), thus, Huh7 cells were not chosen for subsequent experiments. In HCCLM3 cells, depletion of RNF8 by siRNF8 increased sorafenib efficiency when cells grew to 48 or 72 h after sorafenib administration ( Figure 5A,B). In SNU387 cells, RNF8 deficiency by siRNF8-2 induced sensitivity towards sorafenib treatment when cells grew to 72 h, while siRNF8-1 did not reproduce the same results ( Figure 5C,D). In HepG2 cells, knockdown of *RNF8* showed no advantage in enhancing the toxicity of sorafenib ( Figure 5E,F). Thus, inhibition of RNF8 increased sensitivity in liver cancer cells with a poor response to sorafenib but had no impact on cells with a good response, which further supported that RNF8 played a critical role in sorafenib tolerance.

### RNF8 deficiency synergistically sensitizes liver cancer cells to lenvatinib

**Figure 6:**
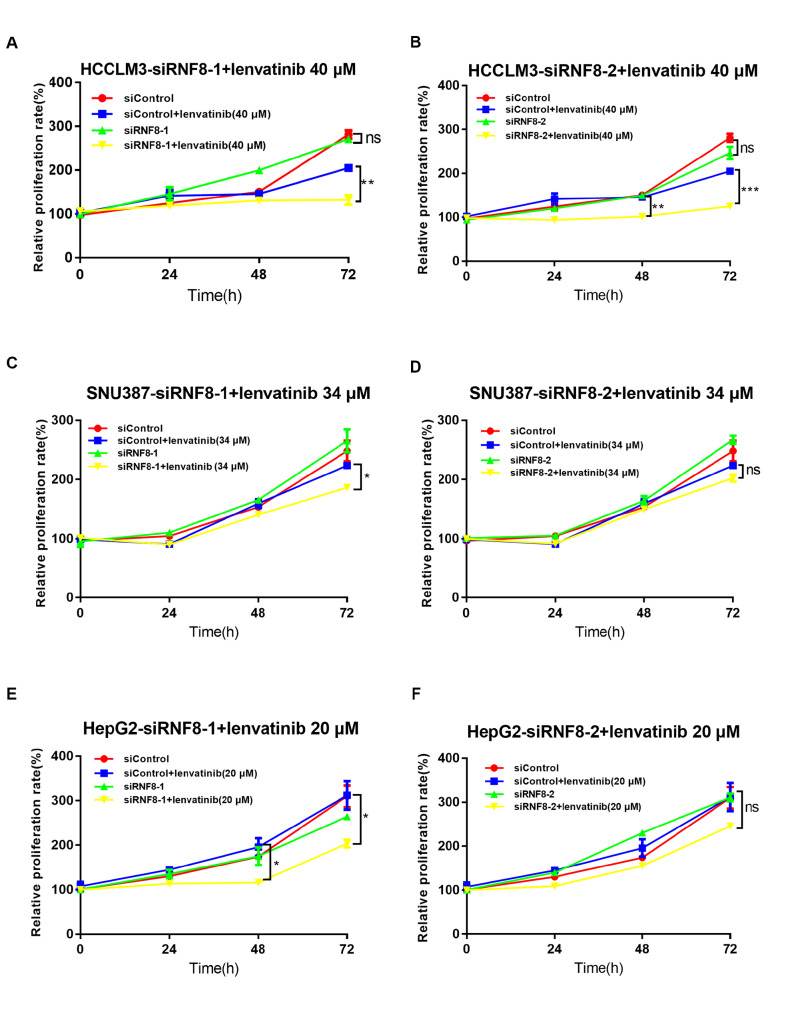
Figure 6 RNF8 deficiency increased the toxicity of lenvatinib in HCC cells Cell proliferation was evaluated via CCK8 assay to detect the effects of RNF8 silencing on lenvatinib toxicity in HCCLM3 (A,B), SNU387 (C,D), and HepG2 (E,F) cells. HCC cells were transfected with the indicated siRNAs and then treated with lenvatinib for 24, 48, and 72 h. * P<0.05, ** P<0.01, *** P<0.001. ns, no significance.

Lenvatinib is another drug approved by the FDA for advanced-stage HCC in the first-line setting with similar benefits to sorafenib but more potent activity against VEGF receptors and the FGFR family [24]. To investigate the possibility that RNF8 increases lenvatinib efficiency, we first detected lenvatinib sensitivity in cancer cell lines and discovered that lenvatinib killed Huh7 cells easily, whereas HCCLM3, HepG2 and SNU387 cells all seemed to be resistant to lenvatinib ( Supplementary Figure S4). Knockdown of *RNF8* by siRNAs was performed to study the role of RNF8 in maintaining lenvatinib resistance. *RNF8*-knockdown HCCLM3 cells exhibited a slower growth rate than cells transfected with siControl under lenvatinib treatment at 48 h or 72 h ( Figure 6A,B). In SNU387 cells, a combination of RNF8 depletion by siRNF8-1 and lenvatinib treatment amplified the inhibitory effects on cell growth compared with lenvatinib alone, although the same effects were not observed in cells transfected with siRNF8-2 and treated with lenvatinib ( Figure 6C,D). Similarly, silencing of *RNF8* by siRNF8-1 enhanced the toxicity to HepG2 cells at 48 and 72 h after lenvatinib administration, although no statistical significance was found for siRNF8-2 ( Figure 6E,F). These data indicated that RNF8 deficiency increased the sensitivity of liver cancer cells to lenvatinib and further confirmed its importance in liver cancer progression and therapies.

## Discussion

Although abundant advances have been achieved in understanding the pathophysiology and drivers of HCC, curative treatments for HCC remain a major problem, especially for advanced patients. Intrahepatic or distant metastasis is the main cause of treatment failure and consequent mortality in HCC patients [25]. Consistent with previous pan-cancer bioinformatics studies [26], we found that RNF8 was upregulated in HCC tissues relative to adjacent non-tumor tissues and that HCC patients with high expression level of RNF8 had shorter survival time, which all indicated the potential of RNF8 in the progression of HCC. Based on these findings, we further revealed that silencing of *RNF8* prevented migration in HCC cells and affected the expressions of EMT regulators such as snail, β-catenin and ZO-1. Moreover, silencing of *RNF8* enhanced the anticancer effects of sorafenib and lenvatinib in HCC cells.

Numerous papers have highlighted the association of the EMT signature with HCC progression, and it was acknowledged that EMT-TFs or upstream signaling regulating these TFs dominated the EMT process in HCC [27]. Here, we found that RNF8 deficiency inhibited the EMT process in HCC cells and resulted in decreased expressions of snail and β-catenin and increased expression of ZO-1. We also revealed that ectopic RNF8 interacted with endogenous β-catenin. Consistent with our findings in this study, previous studies have revealed that RNF8 promotes EMT in lung cancer cells via stabilization of slug and facilitates cancer chemoresistance and progression by triggering K63-linked ubiquitination of Twist [ 19, 28] . Taken together, we speculated that RNF8 might function as a universal regulator of EMT in cancers, and its impacts on the expression of EMT-TFs might be the underlying mechanisms in regulating EMT. However, whether the changes in snail, β-catenin, and ZO-1 expression in HCC are associated with RNF8-mediated ubiquitin modification remains to be further elucidated. Thus, we believe that RNF8 could be an important target for further research in fighting against metastasis in cancers for clinical application.

It has been a long history that EMT is strongly linked to drug resistance [29]. Previous studies have found that drug resistance is related to EMT and EMT inducers such as TGF-β, Wnt/β-catenin, Snail, Slug and ZEB [ 30– 32] . EMT can alter the expressions of ATP-binding cassette (ABC) transporters, promote evasion of apoptosis, and remodel the tumor microenvironment [22], which all contribute to drug resistance in cancer cells. It was also reported that alteration of signaling pathways, including MAPK and Akt/mTOR, could modulate the sensitivity of HCC cells to sorafenib and lenvatinib [ 33– 35] . As expected, RNF8 depletion enhanced the efficiency of sorafenib and lenvatinib in HCC cells. In detail, knockdown of *RNF8* by both siRNF8-1 and siRNF8-2 increased the toxicity of sorafenib and lenvatinib in HCCLM3 cells, while only siRNF8-1 promoted lenvatinib efficacy in HepG2 and SNU387 cells. We proposed that the impacts of RNF8 on drug resistance in HCC cells may be closely associated with its regulation of EMT-TFs. In addition, Xu *et al*. [36] also reported that RNF8 promotes lung cancer cell survival and resistance to DNA damage by regulating AKT. However, we did not find changes in AKT signaling in our experiment; thus, we could not judge whether Akt signaling is involved in RNF8-mediated EMT in HCC cells. For the discrepancy between the two siRNAs of RNF8, we proposed that the difference might be due to their different impacts on the expressions of snail and β-catenin, for siRNF8-2 showed relatively weaker effects on the expressions of these proteins. Notably, depletion of RNF8 alone showed little or no effect on HCC cell proliferation, which further ensured that the enhanced effects on cell growth were due to synergistic effect instead of an additive effect of RNF8 depletion and sorafenib/lenvatinib. Taken together, we proposed that silencing of RNF8 enhances the efficiency of drugs in HCC probably by regulating EMT or other key driver genes.

In summary, we confirmed that knockdown of *RNF8* inhibited EMT and increased drug sensitivity in liver cancer cells, and we also proposed that there was a close relationship between the impacts of RNF8 on EMT and the role of RNF8 in drug sensitivity. We hypothesized that the effects of RNF8 on EMT and drug sensitivity orchestrate its tumor-promoter role in HCC, which could kill two birds with one stone. Therefore, our experiments provide clues for the clinical application of RNF8 as a prognostic marker and a therapeutic target for liver cancer.

## Supporting information

229supplementary_materials

## Supplementary Data

Supplementary data is available at *Acta Biochimica et Biophysica Sinica* online.
