# Supplementary material for: RNF8 depletion attenuates hepatocellular carcinoma progression by inhibiting epithelial-mesenchymal transition and enhancing drug sensitivity: The role of RNF8 in EMT and drug sensitivity in HCC
Source: Acta Biochim Biophys Sin (Shanghai). 2023 May 6;55(4):661–71. doi: 10.3724/abbs.2023076 (PMC10195149; doi:10.3724/abbs.2023076)
Supplement: 229supplementary_materials [file 229supplementary_materials.pdf]

Supplementary Table S1. Clinical information of HCC patients

Patient	Gender	Pathologic diagnosis	HbsAg
Patient 1	male	HCC	+
Patient 2	female	HCC	+
Patient 3	male	HCC	+
Patient 4	male	HCC	+
Patient 5	male	HCC	+
Patient 6	male	HCC	+
Patient 7	female	HCC	+
Patient 8	male	HCC	+
Patient 9	male	HCC	+
Patient 10	male	HCC	+
Patient 11	male	HCC	+
Patient 12	male	HCC	+
Patient 13	male	HCC	+
Patient 14	male	HCC	+
Patient15	male	HCC	+
Patient 16	male	HCC	+
Patient 17	male	HCC	+

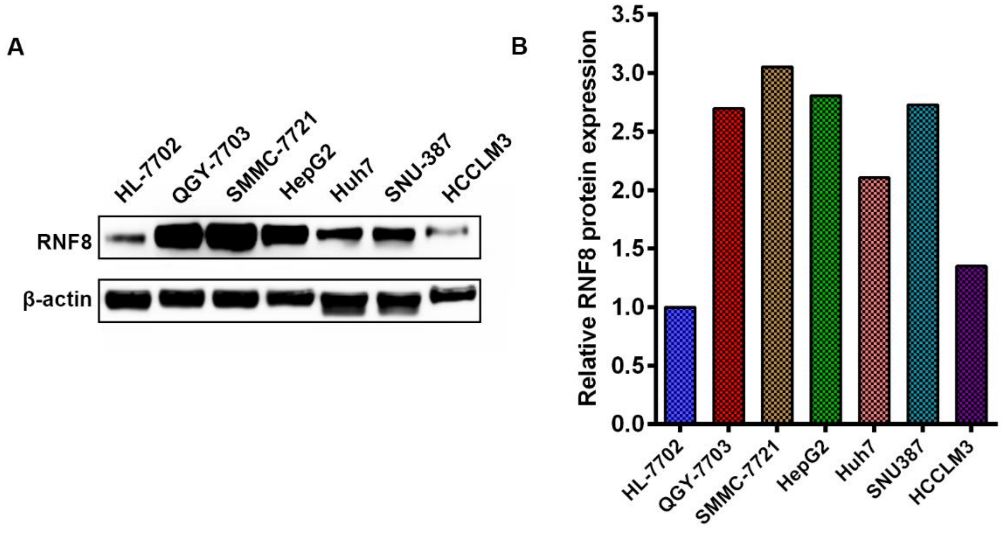

Supplementary Figure S1. Expression of RNF8 in human normal liver cells and liver cancer cells Representative western blots (A) and quantification (B) of RNF8 in normal liver and liver cancer cells. The amount of RNF8 expression was normalized by the level of β-actin.

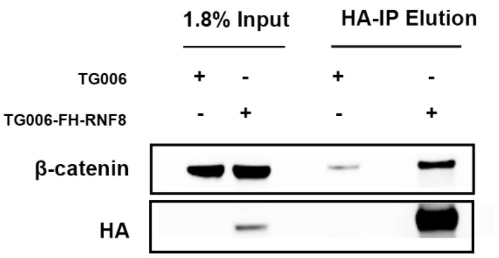

Supplementary Figure S2. RNF8 directly interacts with endogenous β-catenin HEK-293T cells were serially transfected with TG006 or TG006-FH-RNF8. The cells were harvested at 48 h posttransfection, the lysates were coimmunoprecipitated with anti-HA-tag agarose beads, and then subjected to western blot analysis with anti-HA (RNF8) and anti-β-catenin antibodies.

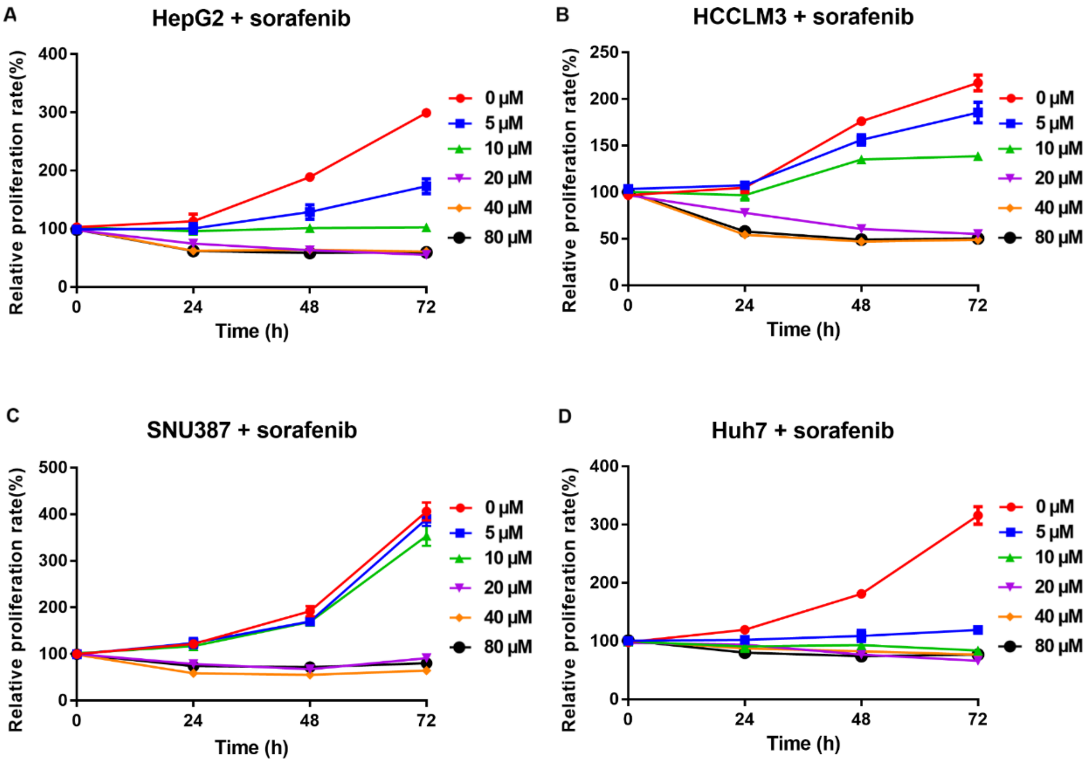

Supplementary Figure S3. Sorafenib inhibits cell growth of HCC cells Relative proliferation rate was measured by CCK8 assay in HepG2 (A), HCCLM3 (B), SNU387 (C), and Huh7 (D) cells under sorafenib administration at 24, 48, and 72 h.

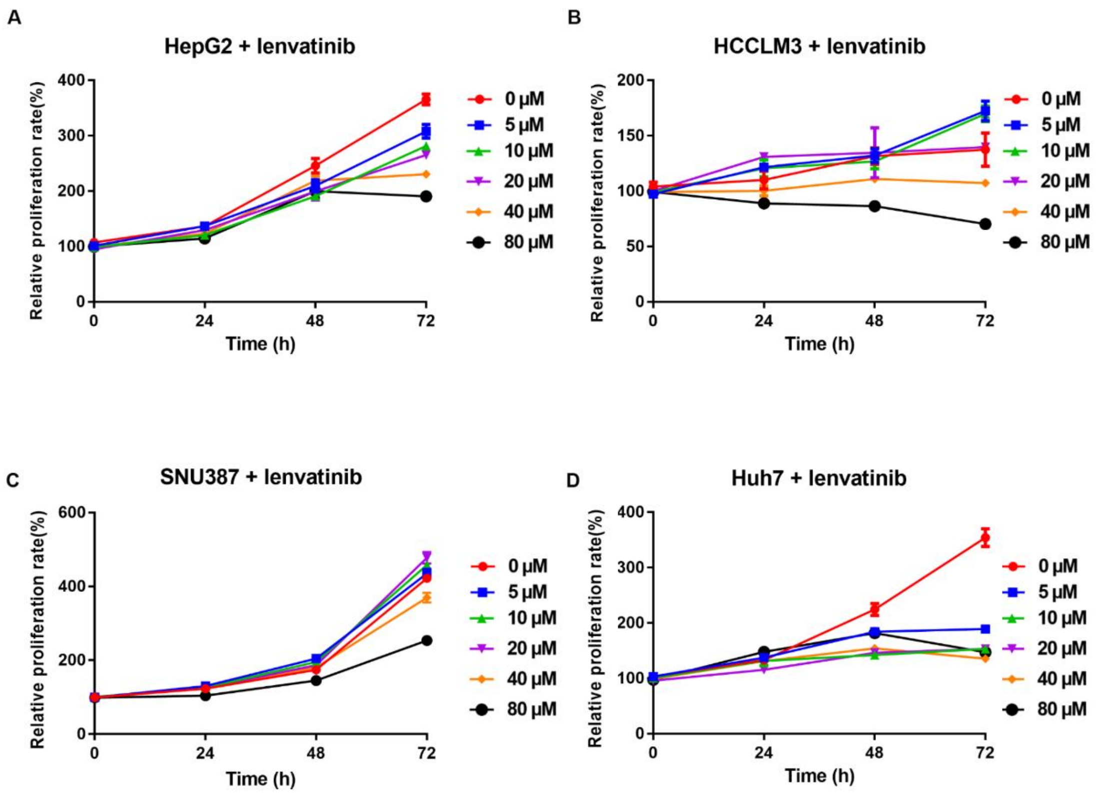

Supplementary Figure S4. Lenvatinib suppresses proliferation of HCC cells Relative proliferation rate showing growth of HepG2 (A), HCCLM3 (B), SNU387 (C), Huh7 (D) cells at 24 , 48, 72 h after lenvatinib treatment.
